# Intervention for Sleep and Pain in Youth (ISPY-RCT): protocol for a two-phase randomized controlled trial of sequenced cognitive-behavioral therapy for insomnia and pain management in adolescents with migraine

**DOI:** 10.1186/s13063-022-07035-9

**Published:** 2023-01-12

**Authors:** Emily F. Law, Lee Ritterband, Chuan Zhou, Tonya M. Palermo

**Affiliations:** 1 Department of Anesthesiology & Pain Medicine, University of Washington School of Medicine, Seattle, WA USA; 2 Center for Child Health, Behavior & Development, Seattle Children’s Research Institute, Seattle, WA USA; 3 Center for Behavioral Health & Technology, University of Virginia School of Medicine, Charlottesville, VA USA; 4 Department of Pediatrics, University of Washington School of Medicine, Seattle, WA USA

**Keywords:** Digital health, Internet interventions, Migraine, Headache, Insomnia, Sleep, Child, Adolescent, Pediatric, Cognitive behavioral therapy

## Abstract

**Background:**

Migraine is a major pediatric health problem impacting 10–12% of youth. About 1 in 3 youth with migraine are diagnosed with insomnia. Sleep and migraine share a cyclical relationship, and data indicate that insomnia symptoms increase migraine severity. CBT for insomnia (CBT-I) has demonstrated efficacy for improving insomnia in adults with migraine and other pain conditions; however, effects in youth have not been evaluated. Moreover, in adults, there is some indication that CBT-I may lead to changes in pain after there are sustained improvements in sleep, but this has never been empirically tested. Cognitive-behavioral therapy for pain management (CBT-Pain) is an established treatment approach for youth with migraine, leading to reductions in headache frequency and disability. In the proposed study, we will address these gaps in knowledge by using an innovative two-phase trial design to (1) test the efficacy of Internet-delivered CBT-I intervention for youth with migraine and comorbid insomnia compared to Internet-delivered sleep education for modifying sleep and (2) investigate how changes in sleep may modify the response to Internet-delivered CBT-Pain intervention.

**Methods:**

We will study a cohort of 180 adolescents, ages 11–17 years, with migraine (with or without aura, chronic migraine) and comorbid insomnia. In phase 1, youth will be randomly assigned to receive Internet-delivered CBT-I intervention or Internet sleep education control. In phase 2, all youth will receive Internet-delivered CBT-Pain intervention. Assessments will occur at baseline, immediately after phase 1 intervention, immediately after phase 2 intervention, and 6 months post-intervention. We will use a comprehensive multidimensional assessment of sleep and headache including self-report questionnaires, ambulatory actigraphy monitoring, and 14-day daily diaries.

**Discussion:**

Given the high prevalence of insomnia in adolescents with migraine, an extension of CBT-I intervention to this population will address an important gap in clinical practice and in conceptual understanding of the relationship between sleep and migraine. By testing a separate CBT-I intervention, we will be able to apply this treatment in the future to other pediatric populations (e.g., cancer, arthritis) who commonly experience comorbid insomnia.

**Trial registration:**

ClinicalTrials.gov NCT04936321. Registered on June 23, 2021.

## Administrative information

Note: The numbers in square brackets in this protocol refer to the SPIRIT checklist item numbers. The order of the items has been modified to group similar items (see http://www.equator-network.org/reporting-guidelines/spirit-2013-statement-defining-standard-protocol-items-for-clinical-trials/).

**Table:** 

Title {1}	Intervention for Sleep and Pain in Youth (ISPY-RCT): protocol for a two-phase randomized controlled trial of sequenced cognitive-behavioral therapy for insomnia and pain management in adolescents with migraine
Trial registration {2a and 2b}.	NCT04936321 [ClinicalTrials.gov] [registered on June 23, 2021].
Protocol version {3}	March 2022, v.1
Funding {4}	This research is funded by the National Institutes of Health/National Institute of Child Health & Development (NIH/NICHD) grant number R01HD101471.
Author details {5a}	E.F. Law: University of Washington School of Medicine & Seattle Children’s Research Institute, Seattle, WA USA L.M. Ritterband: University of Virginia School of Medicine, Charlottesville, VA, USA C. Zhou: University of Washington School of Medicine & Seattle Children’s Research Institute, Seattle, WA USA T.M. Palermo: University of Washington School of Medicine & Seattle Children’s Research Institute, Seattle, WA USA
Name and contact information for the trial sponsor {5b}	Investigator-initiated clinical trial: E.F. Law (principal investigator) emily.law@seattlechildrens.org
Role of sponsor {5c}	This is an investigator-initiated clinical trial. Therefore, the funder played no role in the design of the study; in the collection, analysis, and interpretation of the data; and in the writing of the manuscript.

## Introduction

### Background and rationale {6a}

Migraine is a major pediatric health problem impacting 10–12% of adolescents [1, 2]. Poor sleep is a common comorbidity, with 60–70% of youth reporting elevated insomnia symptoms [3, 4]. Recent studies suggest 30% of adolescents with migraine meet the diagnostic criteria for insomnia [5]. Insomnia is associated with poor outcomes for youth with migraine including greater headache-related disability, more frequent headaches, higher pain intensity, greater depression/anxiety, and greater health service use [4, 6, 7]. There is robust evidence showing the efficacy of psychological treatment for improving headache-related disability and pain in youth with migraine [8, 9]. However, improvements in sleep have been inconsistent [10, 11]. In fact, our preliminary data show that poor baseline sleep is a risk factor for youth to achieve less improvement in pain outcomes with cognitive-behavioral pain treatment (CBT-Pain) [11].

**Fig. 1:**
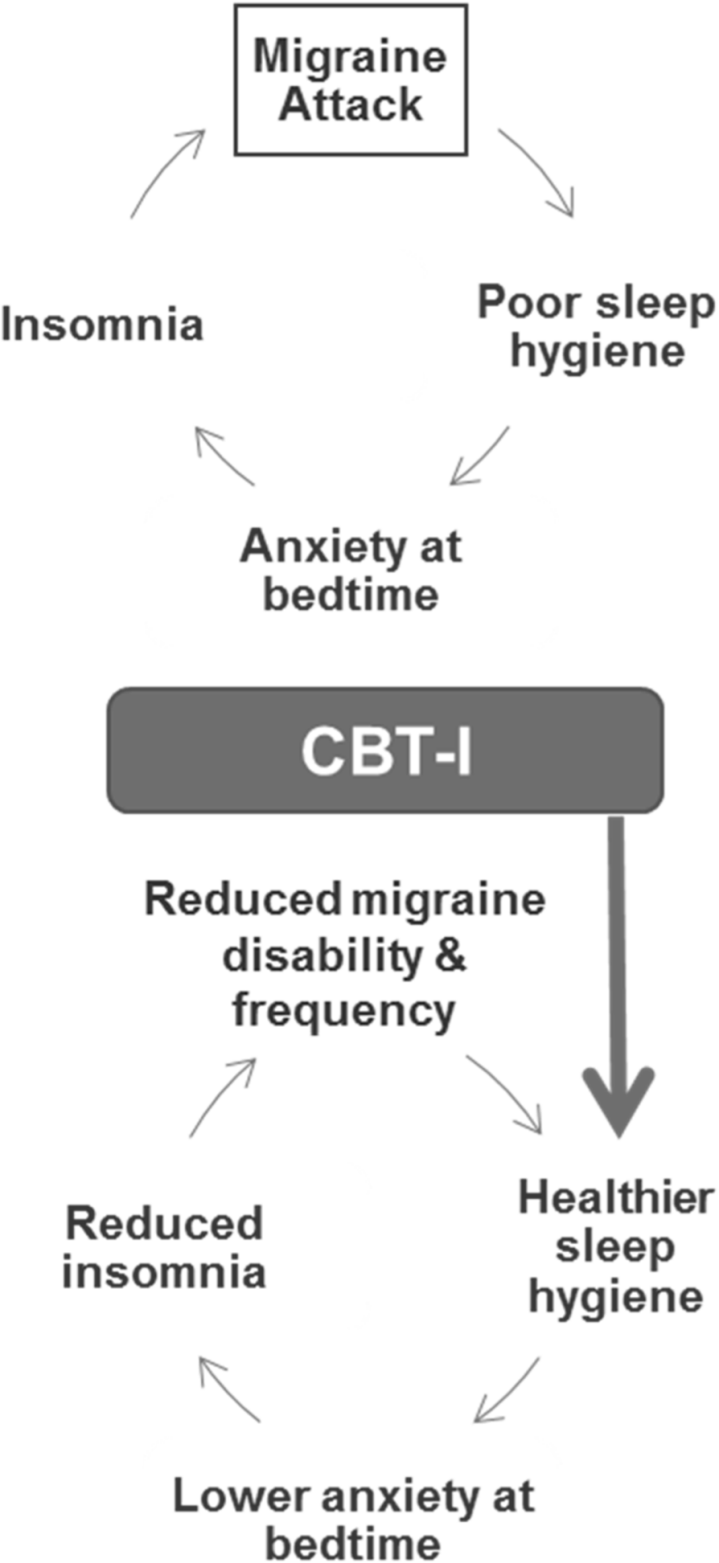
Cycle of migraine and insomnia is disrupted by CBT-I

To cope with migraine, youth commonly engage in maladaptive sleep behaviors (e.g., caffeine overuse, napping, excessive time in bed) that can increase negative cognitions about sleep, disrupt sleep physiology, and lead to a vicious cycle of insomnia symptoms that, in turn, increase the propensity for migraine [12, 13]. We propose that insomnia is a critical treatment target for youth with migraine and that treatment of insomnia could have positive effects on headaches by increasing healthy sleep behaviors and reducing anxiety at bedtime (see Fig. 1 for an illustration of this conceptual model).

Our research team developed a brief cognitive-behavioral treatment for youth with insomnia (CBT-I) delivered in person and demonstrated preliminary efficacy for improving insomnia, sleep quality, sleep hygiene, and sleep patterns in youth with migraine [14] and youth with general physical and psychiatric conditions [15]. Among youth with migraine, we also found improvements in headache-related disability and headache frequency [14]. Although youth and parents found CBT-I to be acceptable, a major barrier to treatment was the requirement of attending in-person treatment visits. Our research team has addressed this treatment barrier in other populations with chronic pain through the use of digital health interventions to deliver CBT-Pain [16, 17]. We expect that this approach will also be effective in delivering insomnia intervention to adolescents with migraine.

### Objectives {7}

The current randomized controlled trial explores the efficacy of Internet-delivered CBT-I intervention (called Firefly) for adolescents with migraine and co-morbid insomnia in a randomized controlled trial using two phases of treatment. In treatment phase 1, the effects of Firefly will be compared to Internet-delivered sleep education control (Sleep EDU) on insomnia symptoms (primary outcome) and sleep quality and sleep patterns (secondary outcomes). In treatment phase 2, both groups will receive Internet-delivered CBT-Pain intervention (called Web-based Management of Adolescent Pain (WebMAP)) to compare the effects of combined insomnia and pain intervention to sleep education and pain intervention on headache-related disability (primary outcome) and headache frequency, pain intensity, anxiety and depressive symptoms, and health-related quality of life (secondary outcomes).

### Trial design {8}

**Fig. 2:**
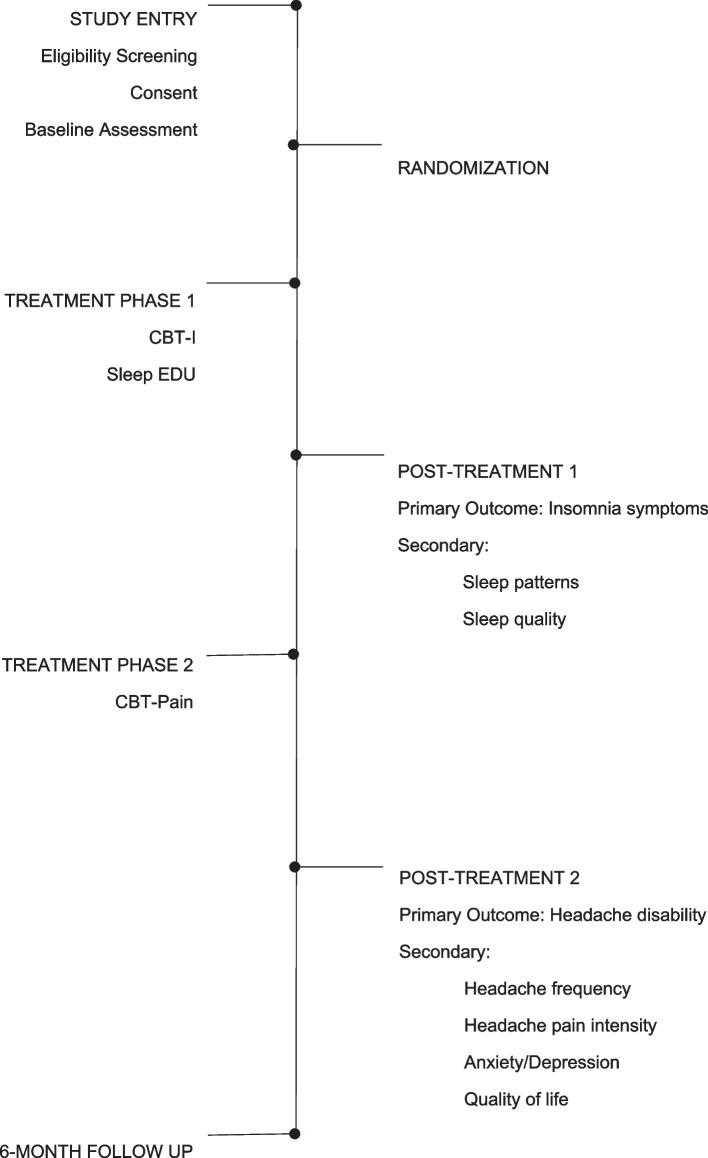
Trial design and study flow

In this two-phase randomized controlled trial with two parallel study arms, we will assess the superiority of CBT-I over Sleep EDU and the superiority of the combined effect of CBT-I + CBT-Pain over Sleep EDU + CBT-Pain. The patient allocation ratio is 1:1. Outcome assessments are administered at baseline, phase 1 post-treatment, phase 2 post-treatment, and 6-month follow-up. See Fig. 2 for an illustration of the trial design and study flow.

## Methods: participants, interventions, and outcomes

### Study setting {9}

Participants will be referred to the study by providers in the Seattle Children’s Hospital Neurology Clinic. This is a tertiary care pediatric neurology clinic in an academic hospital that serves children and families from a 5-state region in the Pacific Northwest, USA.

### Eligibility criteria {10}

We will use a two-stage eligibility screening process. Providers in the Seattle Children’s Hospital Neurology Clinic will identify patients who are 11–17 years old with migraine and self-reported sleep difficulties. Providers will give families a study brochure and submit their contact information to the study team via a secure internal message in the hospital’s electronic health record after obtaining verbal permission to do so from the parent. After receiving referral information, the study staff will contact the families via telephone to conduct a second stage of eligibility screening with the adolescent and parent based on the criteria below. Eligibility criteria were informed by the American Headache Society (AHS) guidelines for trials of behavioral treatments for pediatric headache [18].

Adolescents must meet the following criteria to be eligible for enrollment:

- Age 11–17 years.
- Diagnosis of migraine with or without aura or chronic migraine without continuous headache, as defined by the International Classification of Headache Disorders 3rd Edition (ICHD-3) [19] by the referring provider in Neurology Clinic.
- Meets the research diagnostic criteria for insomnia disorder (self-reported sleep difficulties 3 or more nights during the past month with at least one daytime sleep-related problem) [20].
- Score on the Pediatric Migraine Disability Assessment scale (PedMIDAS) greater than 10, indicating at least mild headache-related disability [21].
- Headache frequency of 6 or more days per month [18].
- Access to the Internet on any web-enabled device.
- Adolescents on a stable dose (× 2 months) of prophylactic medications for migraine or sleep (antidepressants, anticonvulsants, supplements, etc.) will be eligible.

Adolescents who meet any of the following criteria will not be eligible for enrollment:

- Adolescent or parent cannot read in English
- Has another primary sleep disorder (e.g., sleep apnea, narcolepsy) or an unusual sleep/wake schedule or circadian rhythm disorder
- Is currently experiencing a psychiatric crisis (e.g., active suicidality, psychosis)
- Received psychological therapy for insomnia or pain in the 6 months prior to screening

### Who will take informed consent? {26a}

The study staff will conduct informed consent via telephone. Eligible families will provide consent (parents) and assent (adolescents) verbally and in writing using online forms via the Research Electronic Data Capture system (REDCap) [22].

### Additional consent provisions for collection and use of participant data and biological specimens {26b}

N/A: There are no ancillary studies. This trial does not involve collecting biological specimens.

### Interventions

#### Explanation for the choice of comparators {6b}

Adolescents assigned to the control condition in treatment phase 1 receive access to a sleep education website (Sleep EDU) that was developed for this study. The education materials are delivered in six treatment cores, designed to be completed once per week over 6 weeks. The program is metered so that adolescents complete one core per week. Parents receive three email messages (one every 2 weeks) with a summary of the information provided in the adolescent program. The education materials do not include any instruction in cognitive or behavioral skills for insomnia. The content was compiled from publicly available educational websites about sleep (e.g., Kids Health, Sleep Foundation, American Academy of Sleep Medicine). The purpose of this comparator condition is to control for time, attention, and Internet usage and to allow masking of treatment allocation. In our prior RCTs using similar online patient education control conditions, youth and parents have shown a high level of engagement and high ratings of treatment credibility [16].

#### Intervention description {11a}

##### CBT-Insomnia intervention

Adolescents assigned to CBT-I in treatment phase 1 receive access to the Firefly website, an interactive, self-guided intervention that delivers the core components of CBT-I for adolescents. Firefly was adapted from face-to-face CBT-I for adolescents with insomnia and comorbid physical and mental health conditions and Internet-delivered CBT-I for adults [14, 15, 23, 24]. Firefly includes six treatment cores: (1) introduction to the intervention and goal setting, (2) sleep restriction, (3) stimulus control, (4) cognitive skills for sleep, (5) sleep hygiene, and (6) relapse prevention and maintenance. Firefly includes a daily sleep diary which is used to generate tailored recommendations for sleep restriction. Consistent with the core principles of CBT-I, the overarching goal of treatment is to develop a consistent sleep-wake schedule and strengthen the association between bed and sleep by limiting time awake in bed.

**Fig. 3:**
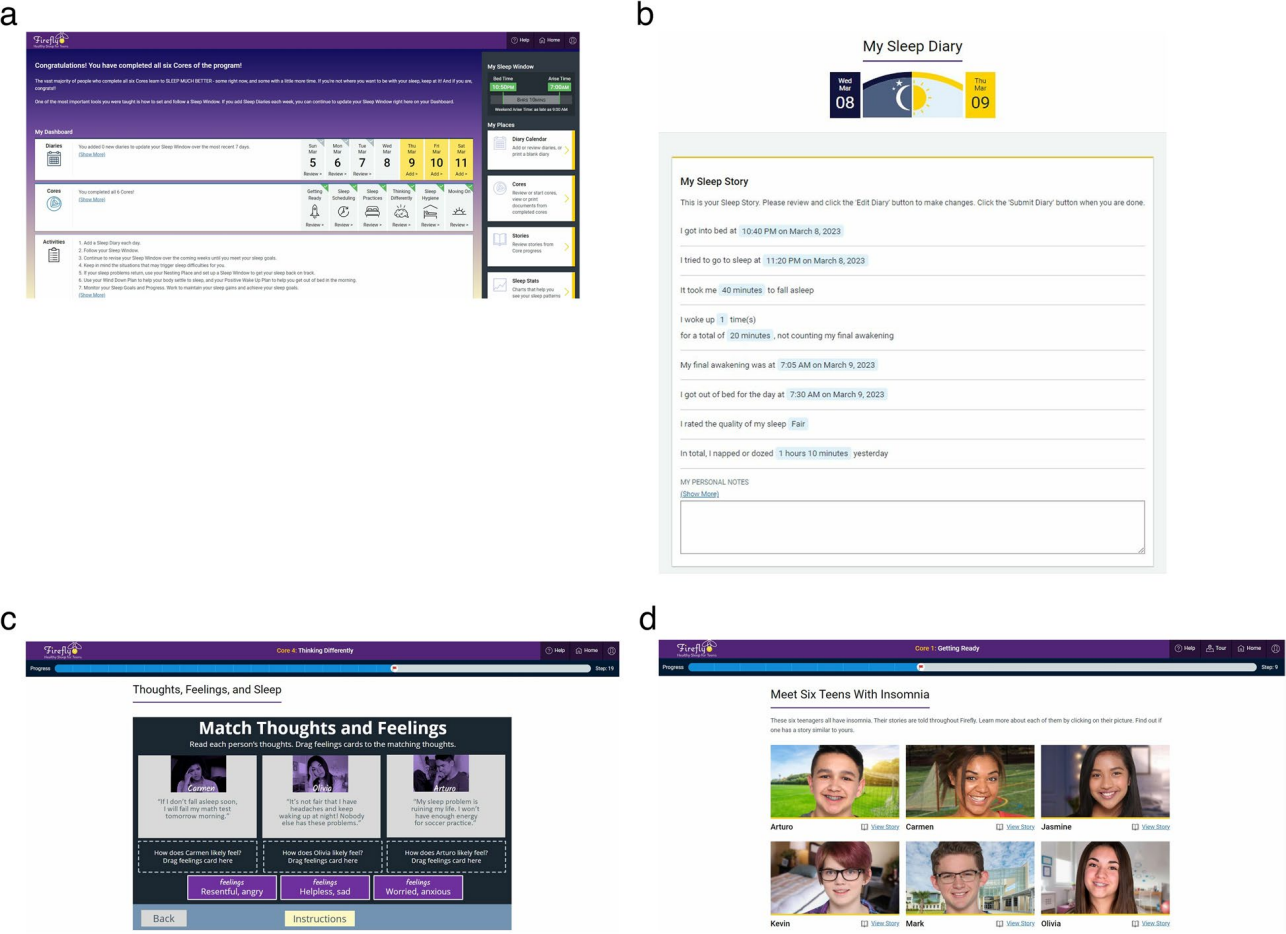
Screenshots from the Firefly website showing **a** home screen, **b** daily sleep diary, **c** skills practice, and **d** peer vignettes

Firefly is metered so that adolescents complete one core per week. Each core includes interactive features to deliver skills training including animations, games, quizzes, and vignettes of peers. At the end of each core, adolescents review their knowledge with brief quizzes and then receive an assignment corresponding with the skills taught in the core (e.g., follow their recommended sleep schedule for sleep restriction, set up a nesting place for stimulus control). Youth are asked to practice the skills in each assignment and complete their daily sleep diary for 7 days, until the next core is released. To support engagement, automated emails are sent when it is time to complete a new treatment core, to enter sleep diaries, and to encourage the completion of homework assignments. See Fig. 3 for screenshots of the Firefly program.

At the beginning of each new core, adolescents report on their experience with the assignment from the prior core and receive tailored recommendations to address barriers and support continued skills practice. Adolescents who have completed at least 5 sleep diaries in the past week also receive personalized recommendations for sleep restriction, which are computed automatically using algorithms developed for the Firefly program with a goal to reach > 85% sleep efficiency with a sleep duration of 9–10 h per night on average. Adolescents who complete fewer than 5 sleep diaries during a given week can move forward when the next core is released but are not provided new recommendations for sleep restriction. Firefly is designed to be completed in 6 weeks. After completing the final core, adolescents can choose to continue using Firefly to monitor their sleep, receive personalized recommendations for sleep restriction, and practice skills as long as they desire.

Consistent with face-to-face CBT-I for adolescents, parents participate in a supportive role and receive a series of 3 email messages (one every 2 weeks) that provide information about the cognitive and behavioral insomnia skills their teen is learning in Firefly and instruction in supporting their teen in using the program and implementing the skills they are learning.

##### CBT-Pain intervention

Treatment phase 2 begins 2–3 weeks after completion of treatment phase 1. In treatment phase 2, all adolescents and parents receive access to WebMAP, an established Internet-delivered CBT-Pain intervention for youth with chronic pain. WebMAP has demonstrated small to moderate treatment effects for improving pain-related disability in multiple clinical trials of youth with chronic pain conditions [16, 17]. WebMAP is guided by cognitive-behavioral, social learning, and family system frameworks [25]. Adolescents and parents access separate versions of the program. The adolescent version of the program includes six treatment cores: (1) pain education and goal setting, (2) cognitive skills for pain management, (3) relaxation methods, (4) coping with pain outside the home, (5) healthy habits, and (6) relapse prevention and maintenance. The parent version of the program also includes six cores: (1) pain education and goal setting, (2) family resilience strategies, (3) operant training, (4) communication skills, (5) modeling and self-care, and (6) relapse prevention and maintenance.

WebMAP is metered so that adolescents and parents complete one core per week. Interactive features include animations, games, quizzes, audio files of relaxation exercises, and video and story vignettes of adolescents and parents coping with pain. At the end of each core, users review their knowledge with brief quizzes and then receive an assignment corresponding with the skills taught in the core (e.g., practice relaxation methods using the audio files). Youth and parents are asked to practice the skills in each assignment for 7 days, until the next core is released. For more details about WebMAP, please see our prior publications [16, 17, 26].

#### Criteria for discontinuing or modifying allocated interventions {11b}

Participants can discontinue the trial procedures and request to be removed from the trial at any time. Study investigators can discontinue a patient’s participation in the study if they do not complete the pre-randomization/baseline assessment, or upon new presentation of exclusion criteria (e.g., inpatient hospitalization, diagnosis of a new serious medical condition). Data that have been collected prior to discontinuation will be included in analyses. This is a low-risk behavioral trial, and adverse events are expected to be minor; therefore, there are no planned criteria for premature study termination or for modifying allocated interventions.

#### Strategies to improve adherence to interventions {11c}

The study staff will interact with participants regularly via text message, email, and telephone to communicate study procedures, encourage program completion, and address general questions and technical issues. Automated email reminders in the Firefly and WebMAP programs will also promote adherence to the intervention. Analytics will be used to track participants’ logins and module completion in the web programs.

#### Relevant concomitant care permitted or prohibited during the trial {11d}

For the duration of the trial, all participants will continue standard care in the neurology clinic. Participants can engage in any other intervention or treatment (e.g., counseling, massage therapy, acupuncture, medication management) as recommended by their care providers. We will collect information on concomitant therapies received during the trial via participant self-report.

#### Provisions for post-trial care {30}

Adverse events are expected to be minor. Therefore, there are no provisions for any additional ancillary or post-trial care or to provide compensation to those who suffer harm from trial participation.

### Outcomes {12}

The primary outcome following treatment phase 1 is change in self-reported insomnia symptoms (Insomnia Severity Index (ISI) [27]) from pre-randomization/baseline to immediately after treatment phase 1, immediately after treatment phase 2, and 6-month follow-up. The ISI has shown strong validity and reliability estimates, including sensitivity to change in response to insomnia treatment for teens with physical and mental health comorbidities [14, 15]. Secondary outcome measures of sleep include self-reported sleep quality (Adolescent Sleep-Wake Scale-Short Form (ASWS-SF)) [28] and sleep patterns (sleep duration, sleep efficiency, and wake after sleep onset) assessed using 14 days of daily sleep diary and actigraphic monitoring (Actiwatch Spectrum Plus, Phillips Respironics, Bend, OR) [29, 30].

The primary outcome following treatment phase 2 is change in activity limitations due to headache (Child Activity Limitations Interview-9 (CALI-9)) [31] from baseline to phase 1 post-treatment, phase 2 post-treatment, and 6-month follow-up. The CALI-9 measures difficulty in performing usual daily physical, social, and recreational activities (e.g., going to school, participating in activities at home) due to headache and has demonstrated sensitivity to change in response to CBT-pain treatment. Adolescents will complete a 14-day electronic diary which includes (1) the CALI-9 diary version [32], (2) the presence of headache (yes/no) to compute headache frequency [33] (secondary outcome), and (3) headache pain intensity (11-point Numerical Rating Scale (NRS)) [34] (secondary outcome). Additional secondary outcomes include self-reported emotional functioning (PROMIS Pediatric Anxiety and Depression short form scales v.2.0) [32] and health-related quality of life (youth self-report Pediatric Quality of Life Inventory 4.0) [35].

Adolescents and parents will complete a Treatment Experience Survey adapted for this study [36] concerning whether they experienced negative effects of study participation (e.g., worrying about sleep or pain, overwhelmed with treatment assignments, tension at home) and rate the level of discomfort experienced (0 = did not affect me/my teen at all, 3 = affected me/my teen very negatively). The number, type, and severity of negative experiences will be summarized. Participants will also report adverse events via free text response at each assessment time point.

### Participant timeline {13}

**Fig. 4:**
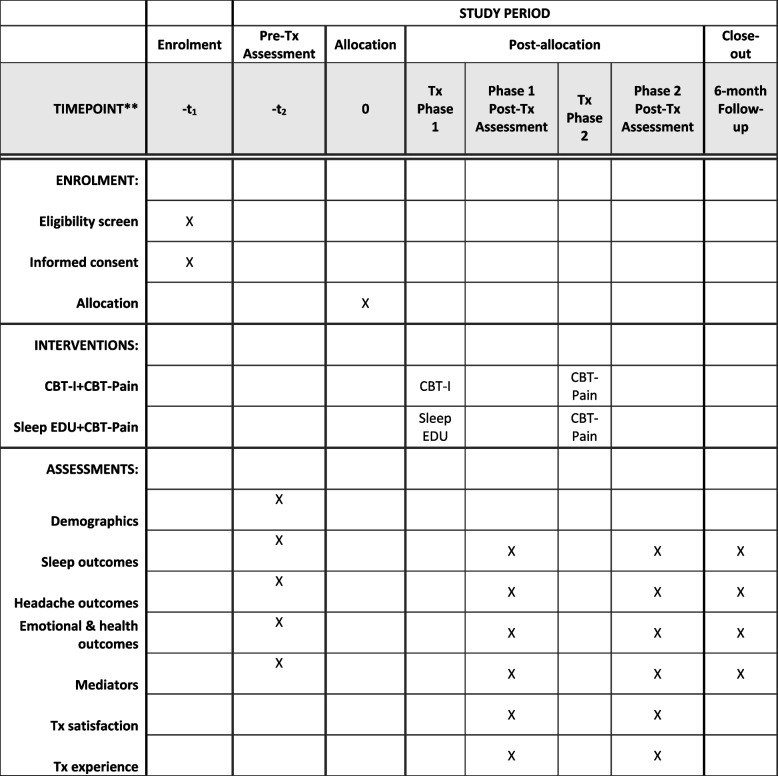
Time schedule of enrollment, interventions, and assessments

Figure 4 shows the participant timeline.

### Sample size {14}

Sample size estimates were based on our team’s preliminary data for CBT-I intervention for adolescents [14, 15], Internet-delivered CBT-I for adults [23, 25], our WebMAP trials [16, 17], and other trials of CBT-Pain in youth with headache or migraine [8, 37, 38]. Based on these preliminary data, power analyses indicated that 77 subjects per group would provide 80% power to detect a moderate effect size (Cohen’s *d* = 0.45) on the primary sleep outcome of insomnia symptoms (ISI [27]) and the primary headache outcome of headache-related disability (CALI-9 [31]). Attrition in our prior web-based trials has been less than 10% [16, 17]. With attrition conservatively estimated at 15%, we expect to enroll 180 subjects to achieve a final sample size of 154 subjects.

### Recruitment {15}

Patients will be recruited from the Seattle Children’s Hospital Neurology Clinic, which carries out over 700 outpatient visits for headaches annually. We estimated an approximate rate of youth with co-morbid migraine and insomnia using data from the 240 participants with migraine recruited into a prior study in this clinic; 71% met the criteria consistent with a positive screen for insomnia [3]. Thus, we conservatively expect 40–50% of referrals to be eligible based on the inclusion and exclusion criteria. In our prior web-based trials, participation rates have been over 50% [16, 17]. Therefore, we anticipate needing to screen 360 patients to recruit 180 patients.

### Assignment of interventions: allocation

#### Sequence generation {16a}

Participants will be randomly allocated to the two study arms in a 1:1 ratio. To further balance the arms, randomization will be blocked with block sizes varying from 4 to 10. An independent data manager not involved in other study procedures will generate the allocation sequence using computer-generated random numbers.

#### Concealment mechanism {16b}

The allocation sequence will be implemented using the REDCap randomization framework [39], which ensures that the allocation sequence remains concealed until interventions are assigned.

#### Implementation {16c}

After completing informed consent, assent, and the pre-treatment assessment, the study staff will use the REDCap randomization framework to allocate the participant to one of the study arms. Different study staff will generate the allocation sequence, conduct participant enrollment, and assign participants to interventions.

### Assignment of interventions: blinding

#### Who will be blinded {17a}

Only the study staff who assigns participants to interventions will be unblinded to the treatment assignment. All other study staff will be blinded to the treatment assignment, including outcome assessors, data analysts, and investigators. This will be achieved by restricting access to participants’ treatment assignments in our tracking database so that it is only viewable by the study staff whose role is to assign participants to interventions. Trial participants and care providers will not be told which intervention arm was assigned to individual patients, so they will also be blinded to treatment assignment.

#### Procedure for unblinding if needed {17b***}***

Unblinding is permissible in the unlikely event that an adverse event occurs. In that case, the study coordinator who assigns participants to interventions would inform the study investigators of the participant’s intervention assignment.

### Data collection and management

#### Plans for assessment and collection of outcomes {18a}

The primary source of data will come from questionnaires and daily diaries which will be completed online using REDCap. Adolescents and parents will complete online assessments privately and independently. Adolescents will also be sent an actiwatch via postal mail to their homes, which they will wear for 14 days and then return to the study staff via postal mail. The study staff will use an electronic database management system to track the administration of assessments. The database will be password protected and stored on a secure server that is only accessible to the study staff. Monthly audits of the database will be conducted to monitor adherence to the study protocol. See Table 1 for the description of the study instruments.

**Table 1:** Primary and secondary outcome measures

	Measure description	Respondent
**Primary outcomes**		
Insomnia symptoms	The 7-item Insomnia Severity Index measures the severity and impact of insomnia symptoms. Items are rated on a 5-point Likert scale (0–4) and summed to create a total score. Total scores above 8 indicate clinically significant insomnia symptoms in adolescents and adults.	Adolescent
Headache disability	The CALI-9 is a validated self-report measure of daily pain-related disability in children and adolescents completed daily for 14 days. The 9-item measure assesses the difficulty in performing usual daily physical, social, and recreational activities due to headache. Total scores range from 0 to 100, with higher scores indicating greater headache-related disability. Average scores over each 14-day measurement period will be computed.	Adolescent
**Secondary outcomes**		
Sleep quality	The Adolescent Sleep Wake Scale-Short Form is a 10-item measure of sleep quality. The total score reflects the overall perception of sleep quality.	Adolescent
Sleep patterns	Minutes of estimated sleep, wake time after sleep onset, and sleep efficiency are calculated from actigraphic monitoring over 14 days with the Actiwatch Spectrum Plus.	Adolescent
Headache frequency	The number of headache days reported each day for 14 days.	Adolescent
Headache pain intensity	11-point numerical rating scale with anchors of 0 = no pain and 10 = worst pain possible. Reported daily for 14 days.	
Anxiety symptoms	The Generalized Anxiety Disorder-7 is a 7-item measure of anxiety symptoms. The total score indicates anxiety severity over the last 2 weeks.	Adolescent
Depressive symptoms	The Patient Health Questionnaire-9 is a 9-item measure of depressive symptoms. The total score indicates the severity of depressive symptoms over the last 2 weeks.	Adolescent
Health-related quality of life	The Pediatric Quality of Life Inventory 4.0 is a measure of health-related quality of life. The total score indicates perceived physical and mental health.	Adolescent

#### Plans to promote participant retention and complete follow-up {18b}

Participants will receive detailed information about the study protocol during recruitment and enrollment and will receive emails, text messages, and telephone reminders from the study staff to complete follow-up assessments. Participants will also be provided with an e-commerce gift card after completing each assessment. Questionnaire measures and daily diaries are completed online so that participants can complete these at a time and place that is convenient for them. All survey responses are saved so that participants do not have to complete questionnaire measures in a single sitting. All participants will be invited to complete follow-up assessments, including those who do not complete the intervention.

#### Data management {9}

##### Questionnaire measures and daily diaries

Questionnaire measures and daily diaries will be collected online via REDCap, which automatically alerts the participant and study staff to missed or implausible responses. To ensure complete data, the study staff will follow up with participants when missing data are identified. Scoring errors will be minimized by the use of syntax to autoscore the questionnaire responses and conduct range checks for data values. The output will be downloaded and stored on our secure server that is only accessible to the study staff.

##### Actigraphy monitoring

Adolescents will complete 14 days of actigraphy monitoring to assess sleep patterns using the Actiwatch Spectrum Plus (Philips Respironics, Bend, OR, USA), a watch-like device worn on the non-dominant wrist that records sleep-wake patterns based on movement detected by an omnidirectional sensor. Participants will also (a) push an event marker on the Actiwatch at bedtime and when they wake in the morning and (b) complete a corresponding daily electronic sleep diary each morning to report on the prior night’s sleep pattern (bedtime, wake time, number and duration of night wakings). To ensure data quality, a standardized actigraphy scoring protocol will be followed. Data will be scored in 1-min epochs using the Philips Actiware version 6.2.0 software (Philips Respironics, Bend, OR, USA). To minimize scoring errors and ensure the validity of actigraphy data, event markers and sleep diary data will be used to assist with scoring decisions.

Three actigraphy variables will be calculated for analyses: (1) total sleep time—the total amount of time scored as sleep in minutes from sleep onset to sleep offset, with sleep onset defined as the first 10-min segment with no more than one epoch of any recorded activity and sleep offset defined as the last 10-min segment with no more than one epoch of any recorded activity; (2) wake minutes after sleep onset—the number of minutes scored as wake after nighttime sleep onset; and (3) sleep efficiency—the ratio of total sleep time and total time spent in bed at night as a percentage, with values closer to 100 indicating more efficient sleep [30].

#### Confidentiality {27}

Contact information for potential participants will be sent by referring providers to the study staff via a secure internal message in the Seattle Children’s Hospital electronic health record. Identifying information and research data will be stored separately on encrypted, secure, password-protected databases on Health Insurance Portability and Accountability Act (HIPPA)-compliant servers at Seattle Children’s Research Institute. Research data will be coded with a unique identifier, and no personal identifiers will be present among research data. The code that links identifying information to study identifiers will be accessible only to the study staff. All research data will be deidentified at the earliest possible opportunity to promote data sharing.

#### Plans for collection, laboratory evaluation, and storage of biological specimens for genetic or molecular analysis in this trial/future use {33}

N/A; biological specimens will not be collected in this study.

## Statistical methods

### Statistical methods for primary and secondary outcomes {20a}

Given the 2-phase RCT design, group comparisons for sleep outcomes will be assessed at phase 1 post-treatment. We will compare insomnia symptoms (primary outcome), sleep quality, and sleep patterns and their corresponding changes from baseline to phase 1 post-treatment between CBT-I and Sleep EDU using the Student *t*-tests or chi-squared tests. Baseline demographic and pre-treatment variables are expected to be balanced across treatment conditions by randomization but will be checked. We will conduct covariate-adjusted regression analysis on the outcomes: linear regression models on continuous outcomes and logistic regression models on binary outcomes, with a priori identified biological covariates (age, sex), sociodemographic variables (race, ethnicity), and baseline outcome values. The predictor of interest is the treatment condition indicator. All regression models will be carefully assessed using residual analysis and goodness-of-fit statistics (deviance, likelihood ratio, information criteria, etc.). The primary hypotheses will be tested using the Wald test on the coefficient estimates of treatment condition indicator.

Group comparisons for headache outcomes and emotional and health outcomes will be assessed at phase 2 post-treatment and 6-month follow-up. Similar analyses as outlined for phase 1 outcomes above will be applied for cross-sectional analyses of post-treatment phase 2 outcomes. Longitudinal data analysis will examine the sleep and headache outcome variables assessed at all 4 time points (baseline, phase 1 post-treatment, phase 2 post-treatment, 6-month follow-up). All regression models will have the following form: outcome ~ group + time + time × group + confounders. We will model time as a discrete variable. The differences in changes from baseline in outcomes between the treatment groups will be assessed by testing the significance of coefficients for the time-by-group interaction terms using the Wald test (individual) and likelihood ratio test (overall). To account for clustering due to repeated assessments within individuals, we will use linear and generalized linear mixed effects regression models.

### Interim analyses {21b}

There are no interim analyses planned.

### Methods for additional analyses (e.g., subgroup analyses) {20b}

We will examine the effect of treatment adherence on primary and secondary outcomes. We will create a treatment exposure variable (number of completed treatment modules) and test the significance of coefficients for the time-by-treatment exposure interaction terms.

### Methods in analysis to handle protocol non-adherence and any statistical methods to handle missing data {20c}

Primary analyses will be intent-to-treat analyses, which will include all participants randomized regardless of intervention completion. Missing data will be minimized to the extent possible using the methods described above. If needed, for multi-item instruments, missing data at the item level will be addressed using published guidelines where available. For primary and secondary outcome analyses, the amount of missing data and pattern of missing data will be examined. If a substantial amount of missing data exists, in addition to our primary data analysis using all available data, we will conduct additional sensitivity analysis using multiple imputations with changed equations.

### Plans to give access to the full protocol, participant-level data, and statistical code {31c}

The investigators will share data on a case-by-case request.

### Oversight and monitoring

#### Composition of the coordinating center and trial steering committee {5d}

This is a single-site study designed, performed, and coordinated at Seattle Children’s Hospital and Seattle Children’s Research Institute. The study team meets weekly. There is no steering committee or adjudication committee. Day-to-day support for the trial is provided by the following:

- Principal investigator: provides oversight for all aspects of the trial, including consenting and assenting procedures, maintenance of IRB approval, intervention implementation, and data management
- Data manager: organizes data capture and ensures data quality
- Study coordinators: conduct recruitment, administer informed consent and assent, manage IRB submissions and annual renewal reports, and administer assessment and intervention procedures according to protocol

#### Composition of the data monitoring committee, its role, and reporting structure {21a}

In agreement with the Seattle Children’s Research Institute IRB and the NIH/NICHD, a DSMB has not been appointed for this study. This study is not considered a NIH-defined phase III clinical trial. Furthermore, the assessments and behavioral interventions have been used previously by the study investigators in our prior research and clinical work. The risks of the interventions are known, and they are minimal. Moreover, participation in this study is adjunctive to routine treatment through the Seattle Children’s Hospital Neurology Clinic, and thus, participants will be monitored by their treating physicians. Treatment in the neurology clinic will not be altered by study participation. A neurologist knowledgeable in the field of pediatric headache and insomnia and a biostatistician knowledgeable in pediatric clinical trials will be assigned as data and safety officers for this study. In the case of suspected adverse events, they will be contacted to assess whether the suspected adverse event is related to treatment and will recommend the next steps for safety measures when indicated.

#### Adverse event reporting and harms {22}

The study staff will have frequent interactions with participants (1–2 times per week) during the intervention period to monitor program completion and to evaluate any unwanted treatment reactions or adverse events. In addition, at each of the post-treatment assessment time points, participants will complete the Treatment Experiences Survey which is a self-report scale that assesses unwanted changes in sleep, pain, and stress as a result of study participation. Responses that indicate a potential adverse event or unanticipated event that may be related to the study protocol will be escalated to the PI within 48 h of identification and will be reviewed with the study’s data and safety officers. Adverse events that are determined to be a result of study participation will be reported to the IRB and the funding agency (NIH/NICHD). In the event that a participant withdraws from the study or is discontinued due to a serious adverse event, the participant will receive follow-up monitoring by the study staff until the problem has been resolved or stabilized or is determined to be unrelated to the study. All adverse events, serious and non-serious, will also be reported annually to the IRB during the yearly renewal process.

#### Frequency and plans for auditing trial conduct {23}

The data manager will conduct internal monthly audits for 10% of randomly selected participants to monitor conformance with informed consent requirements, completeness of study records, and verification of source documents. Auditing can also take place by the Seattle Children’s Hospital IRB.

#### Plans for communicating important protocol amendments to relevant parties (e.g. trial participants, ethical committees) {25}

Any protocol modifications will be updated on ClinicalTrials.gov and in the participants’ consent forms.

#### Dissemination plans {31a}

Trial results will be communicated via publication in peer-reviewed journals, reporting on ClinicalTrials.gov, and presentations at professional society meetings. Participants will receive a summary of the trial results in newsletter format.

## Discussion

This randomized controlled trial is designed to investigate the efficacy of Internet-delivered CBT-I compared to Internet-delivered Sleep EDU in 180 adolescents with migraine and insomnia. The potential synergistic effects of delivering CBT-I followed by CBT-Pain on headache outcomes and emotional and health outcomes will also be examined by comparing CBT-I+CBT-Pain to Sleep EDU+CBT-Pain.

### Limitations

There are several limitations to consider. First, the study protocol may be time intensive which might impair enrolment or increase drop-out. The risk of drop-out will be minimized by informing potential participants of the study protocol during the recruitment and enrolment process. Second, the comparison of CBT-I to Sleep EDU is limited to the immediate phase 1 post-treatment assessment at phase 1, and subsequent assessment time points will reflect the addition of CBT-Pain to either CBT-I or Sleep EDU. We chose the 2-phase trial design to provide information about the potential synergistic effects of sleep and pain interventions. Finally, this is a single-site study of youth recruited from a tertiary care pediatric neurology clinic. Our findings may not generalize to patients seen in other settings.

### Strengths

The results of this study will provide the first rigorous RCT data on the treatment of comorbid migraine and insomnia in adolescents. Furthermore, the innovative 2-phase design allows for testing the impact of insomnia intervention compared to control in the first phase while then allowing a comparison of insomnia and pain intervention to pain intervention alone in the second phase. These data are critical for understanding the potential limitations (or benefits) of CBT-Pain interventions in youth with migraine and comorbid sleep problems can help determine whether CBT-I intervention has the potential to improve response to CBT-Pain treatment in this population and can further our conceptual understanding of the sleep-pain relationship.

Using the Internet to deliver treatment is also a strength by providing a scalable intervention that has the potential to be widely disseminated. Because the development and evaluation of insomnia interventions in pediatrics have lagged far behind adult therapies, we developed the Firefly intervention as a stand-alone intervention for insomnia. This provides a future opportunity to extend the Firefly program to a wide spectrum of youth with insomnia, such as those with other health or mental health conditions (e.g., cancer, arthritis, major depression) who are also at high risk for comorbid insomnia.

### Trial status

Recruitment started in October 2021. The currently approved protocol version is 1.00 (initial submission approval 10/07/2022). Recruitment is estimated to be completed in October 2023.

## Data Availability

Any data required to support the protocol can be supplied on request.

## Abbreviations

CALI-9: Child Activity Limitations Interview-9 item
CBT-I: Cognitive-behavioral therapy for insomnia
CBT-Pain: Cognitive-behavioral therapy for pain management
DSMB: Data Safety Monitoring Board
HIPPA: Health Insurance Portability and Accountability Act
ISI: Insomnia Severity Index
PedMIDAS: Pediatric Migraine Disability Assessment
PROMIS: Patient Reported Outcomes Measurement Information System
REDCap: Research Electronic Data Capture
Sleep EDU: Sleep education control
WebMAP: Web-based Management of Adolescent Pain
